# Successful endoscopic submucosal dissection for a cecal laterally spreading tumor using a motorized spiral enteroscope in a case with difficult intubation

**DOI:** 10.1055/a-2078-1165

**Published:** 2023-05-10

**Authors:** Kurato Miyazaki, Motohiko Kato, Kaoru Takabayashi, Noriko Matsuura, Naohisa Yahagi

**Affiliations:** 1 Division of Gastroenterology and Hepatology, Department of Internal Medicine, Keio University School of Medicine, Tokyo, Japan; 2 Division of Research and Development for Minimally Invasive Treatment, Cancer Center, Keio University School of Medicine, Tokyo, Japan; 3 Center for Diagnostic and Therapeutic Endoscopy, Keio University School of Medicine, Tokyo, Japan

In colorectal endoscopic submucosal dissection (ESD), it is essential to intubate with the endoscope in a straight position and maintain stable maneuverability during the procedure. It is however often difficult to obtain these good conditions, especially in patients with colon elongation, poor fixation, or severe obesity. Recently, a motorized spiral enteroscope (MSE) was launched 1 2 3 ( Fig. 1 ). MSE enables deep intubation and appropriate fixation by rotation of the fins around the scope to squeeze the intestinal tract. Here, we report a very challenging cecal ESD in which the MSE was extremely useful ( Video 1 ).

**Fig. 1:**
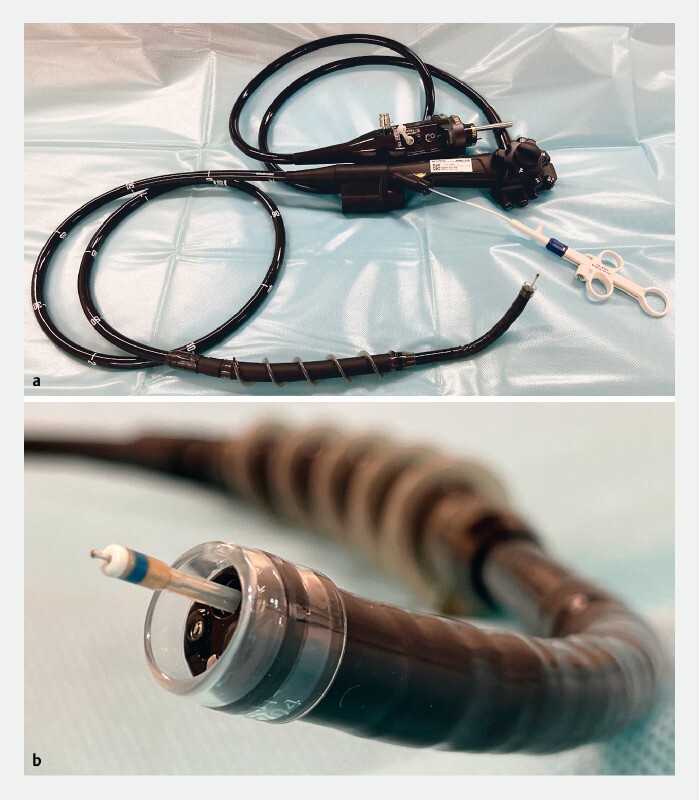
Photographs showing the motorized spiral enteroscope with an energy device for colorectal endoscopic submucosal dissection delivered through the working channel: **a** full view; **b** close up of the tip (it can be equipped with a hood, can deliver energy devices, and has waterjet function capability).

**Video 1** Successful endoscopic submucosal dissection is performed using a motorized spiral enteroscope for a cecal laterally spreading tumor in a patient where intubation was difficult.


A severely obese 71-year-old man (body mass index 38 kg/m ^2^ ) was found to have a 50-mm laterally spreading tumor in the cecum. Because cecal intubation was very difficult and ESD was considered very challenging, he was referred to our hospital. At first, we attempted ESD with a single-balloon endoscope, but we were unable to reach the lesion sufficiently after trying for more than 90 minutes. Therefore, we decided to perform ESD with an MSE.

We achieved successful cecal intubation in 13 minutes, without any difficulty. Thereafter, we performed ESD using the water pressure method 4 5 ( Fig. 2 ). Although it was difficult to approach the distal side of the lesion, we were able to overcome this difficulty by suctioning the air and appropriately using forward rotation. The stable maneuverability of the scope was maintained and the patient did not complain of discomfort during the procedure. Consistent with this, intraoperative abdominal radiography showed that there was no migration of gas into the downstream colon, and the straight scope position was maintained ( Fig. 3 ). En bloc resection was achieved in 62 minutes, without any adverse events ( Fig. 4 ).

**Fig. 2:**
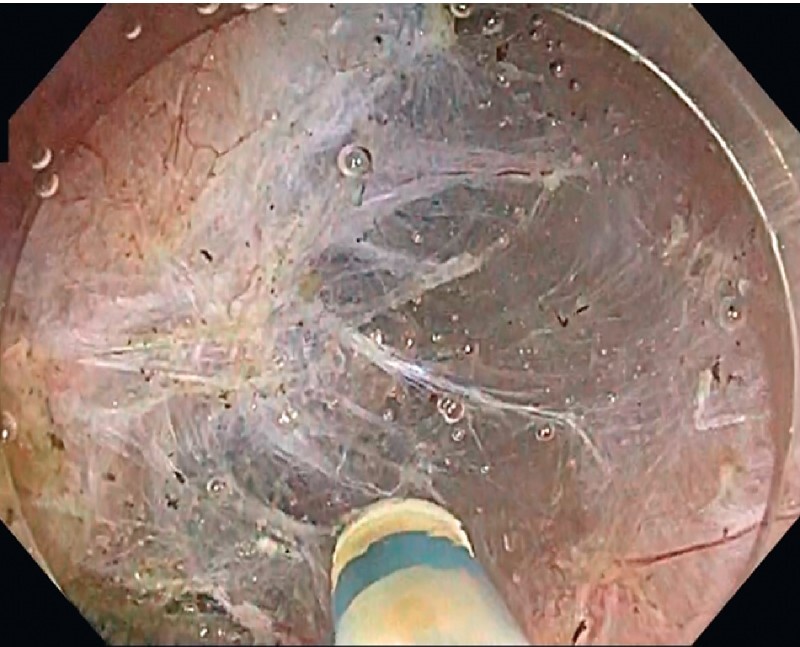
White-light image during endoscopic submucosal dissection using the water pressure method, which was possible because of the stability and maneuverability of the scope.

**Fig. 3:**
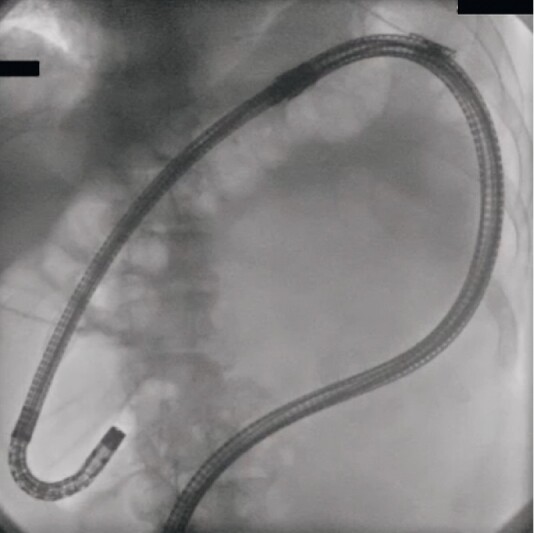
Abdominal radiograph during endoscopic submucosal dissection showing no migration of gas into the downstream colon, and a straight position of the scope.

**Fig. 4:**
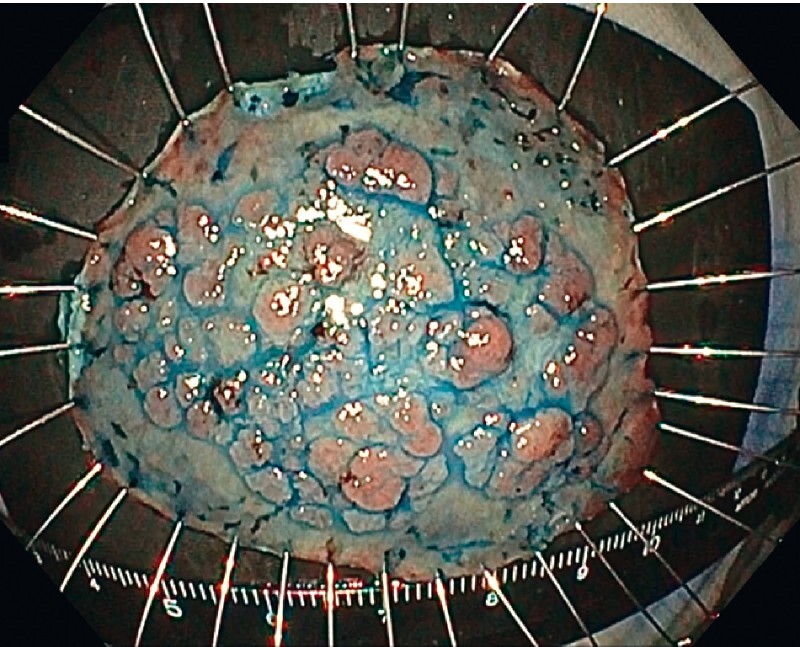
White-light photograph of the specimen stained with indigo carmine after successful R0 resection.

MSE can overcome difficulties in challenging ESD procedures such as this by improving maneuverability, as well as aiding deep intubation into the small intestine, for which it was originally developed.

Endoscopy_UCTN_Code_TTT_1AO_2AG

## Notes

Endoscopy E-Videos

https://eref.thieme.de/e-videos
*E-Videos* is an open access online section of the journal *Endoscopy* , reporting on interesting cases and new techniques in gastroenterological endoscopy. All papers include a high-quality video and are published with a Creative Commons CC-BY license. Endoscopy E-Videos qualify for HINARI discounts and waivers and eligibility is automatically checked during the submission process. We grant 100% waivers to articles whose corresponding authors are based in Group A countries and 50% waivers to those who are based in Group B countries as classified by Research4Life (see: https://www.research4life.org/access/eligibility/ ). This section has its own submission website at https://mc.manuscriptcentral.com/e-videos
